# Supplementary figures and images for: HillTau: A fast, compact abstraction for model reduction in biochemical signaling networks
Source: PLoS Comput Biol. 2021 Nov 29;17(11):e1009621. doi: 10.1371/journal.pcbi.1009621 (PMC8659295; doi:10.1371/journal.pcbi.1009621)

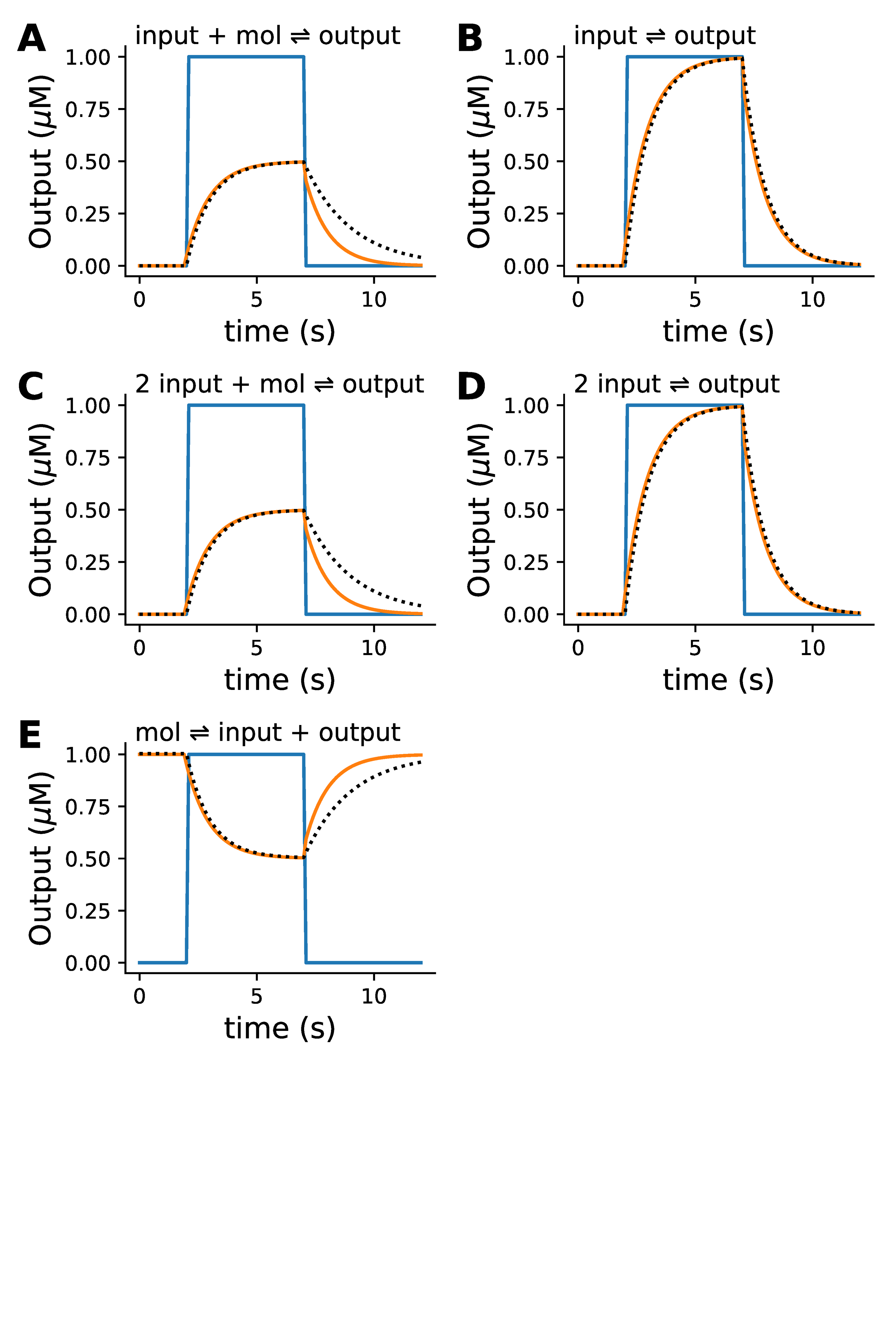

Supplement: S1 Fig — Fits are indicated on top of each figure panel. Each of these is a single HillTau ‘reaction’ where ‘input’ is activator in all but Panel E, where ‘input’ is an inhibitor. In all cases the rising phase fits exactly, but in panels A, C and E the falling phase has a different time-course. (TIF) [file pcbi.1009621.s001.tif]

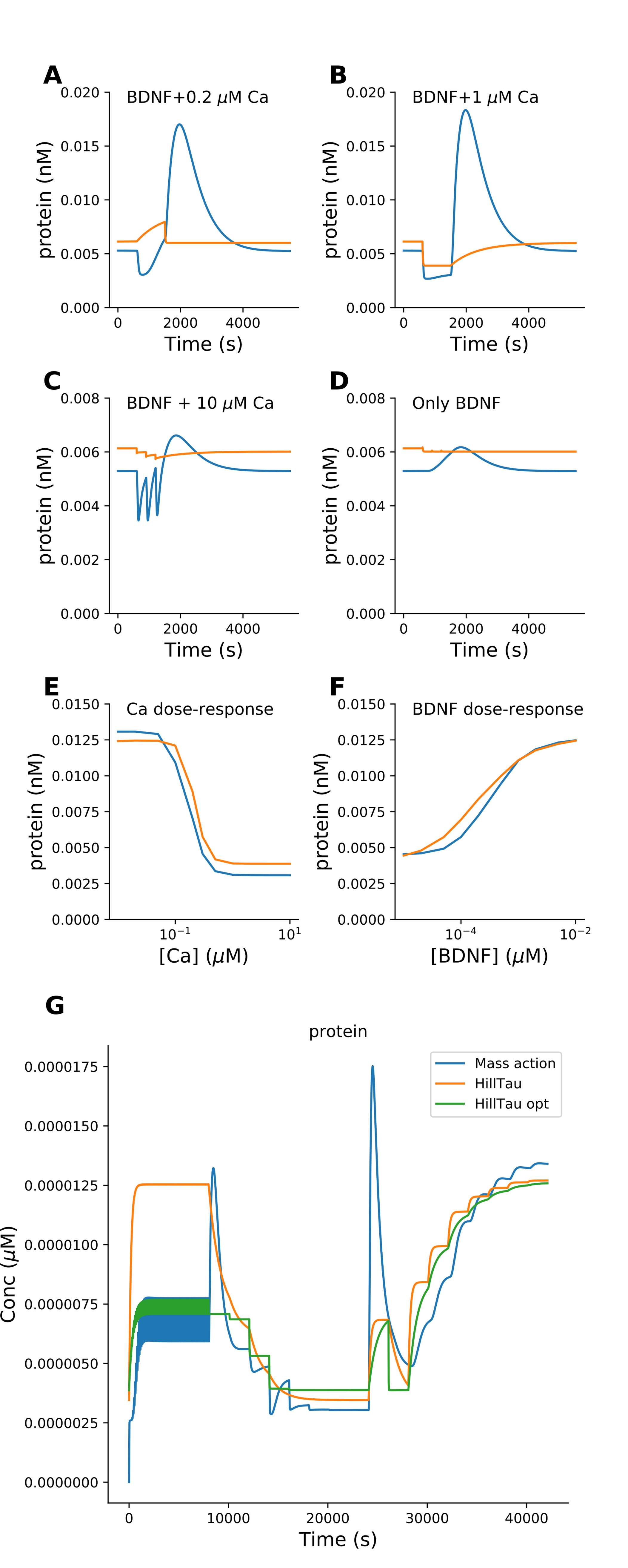

Supplement: S2 Fig — Model is as in Fig 5B. Panels A-F correspond to panels D-I in Fig 5. In all cases protein production rate is readout. Blue plots are reference, orange areHillTau. A: BDNF@3.7 nM + Ca2+@0.2 μM, 900 seconds. B: BDNF@3.7nM, Ca2+@1μM. C: 3 pulses of BDNF@3.7 nM for 5s, coincident with Ca2+@10μM for 1s, pulses separated by 300 s. D: Same as C, but Ca2+ held at baseline of 0.08 μM. E: Dose-response of protein vs. Ca2+, holding BDNF fixed at 3.7 nM. F: Dose-response of protein vs BDNF, holding Ca2+ fixed at 0.08 μM. G: MASH optimization waveform used to fit the HillTau model for protein synthesis. (TIF) [file pcbi.1009621.s002.tif]

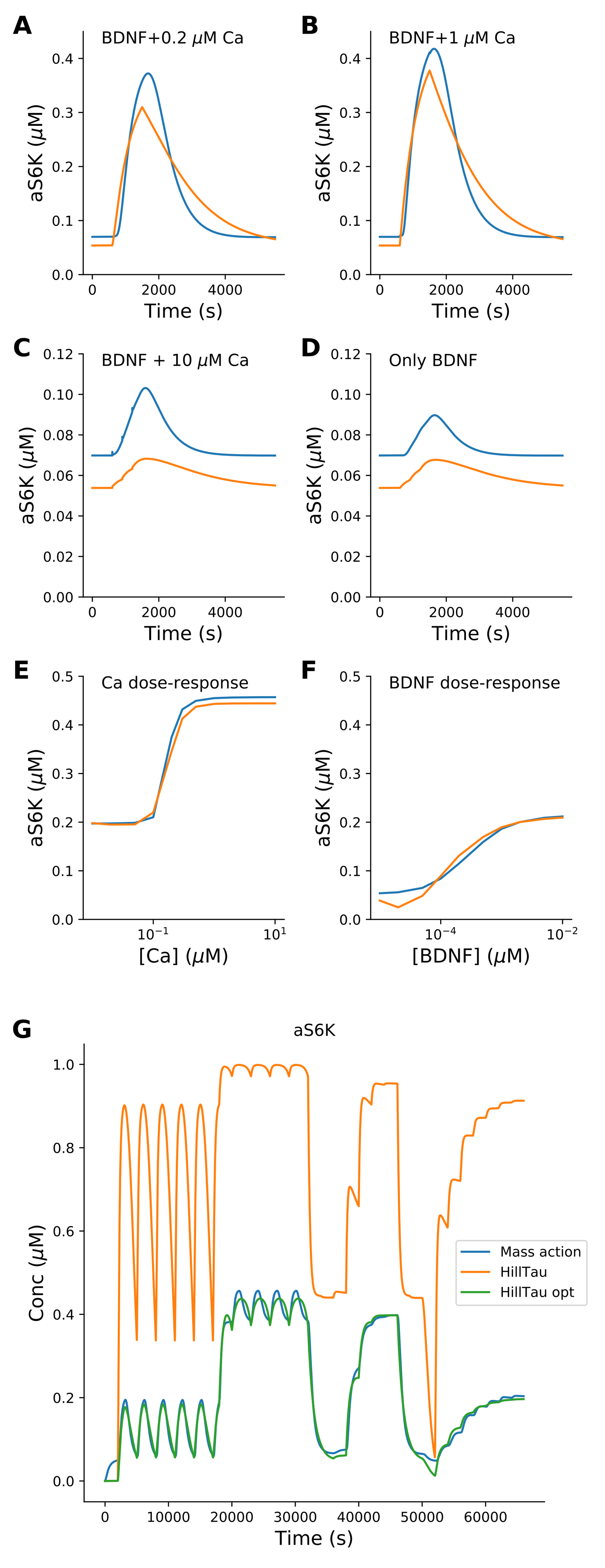

Supplement: S3 Fig — HillTau reactions as in Fig 5C. Panels A-F correspond to panels D-I in Fig 5. In all cases activated S6K concentration is readout. Blue plots are reference, orange areHillTau. A: BDNF@3.7 nM + Ca2+@0.2 μM, 900 seconds. B: BDNF@3.7nM, Ca2+@1μM. C: 3 pulses of BDNF@3.7 nM for 5s, coincident with Ca2+@10μM for 1s, pulses separated by 300 s. D: Same as C, but Ca2+ held at baseline of 0.08 μM. E: Dose-response of protein vs. Ca2+, holding BDNF fixed at 3.7 nM. F: Dose-response of protein vs BDNF, holding Ca2+ fixed at 0.08 μM. G: MASH optimization waveform used to fit the HillTau model for S6K activation. (TIF) [file pcbi.1009621.s003.tif]

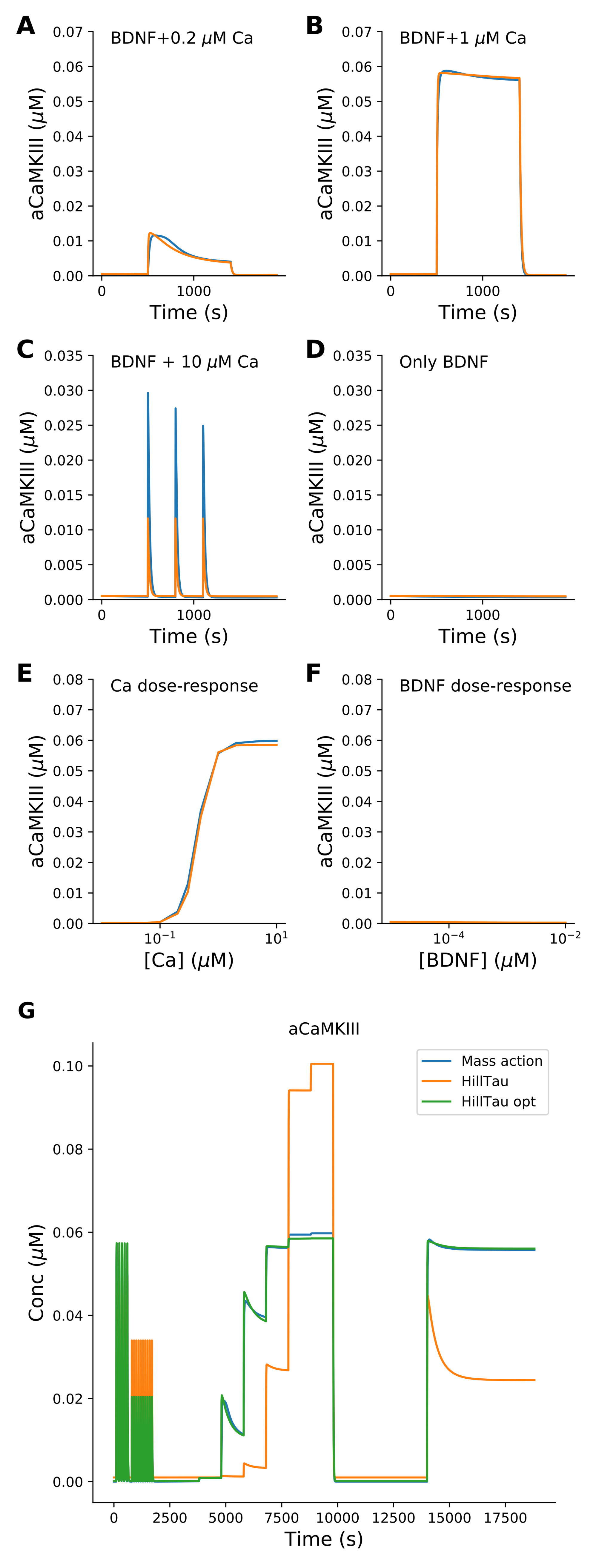

Supplement: S4 Fig — HillTaureactions as in Fig 5C. Panels A-F correspond to panels D-I in Fig 5. In all cases activated CaMKIII concentration is readout. Blue plots are reference, orange areHillTau. A: BDNF@3.7 nM + Ca2+@0.2 μM, 900 seconds. B: BDNF@3.7nM, Ca2+@1μM. C: 3 pulses of BDNF@3.7 nM for 5s, coincident with Ca2+@10μM for 1s, pulses separated by 300 s. D: Same as C, but Ca2+ held at baseline of 0.08 μM. E: Dose-response of protein vs. Ca2+, holding BDNF fixed at 3.7 nM. F: Dose-response of protein vs BDNF, holding Ca2+ fixed at 0.08 μM. G: MASH optimization waveform used to fit the HillTau model for CaMKIII activation. (TIF) [file pcbi.1009621.s004.tif]

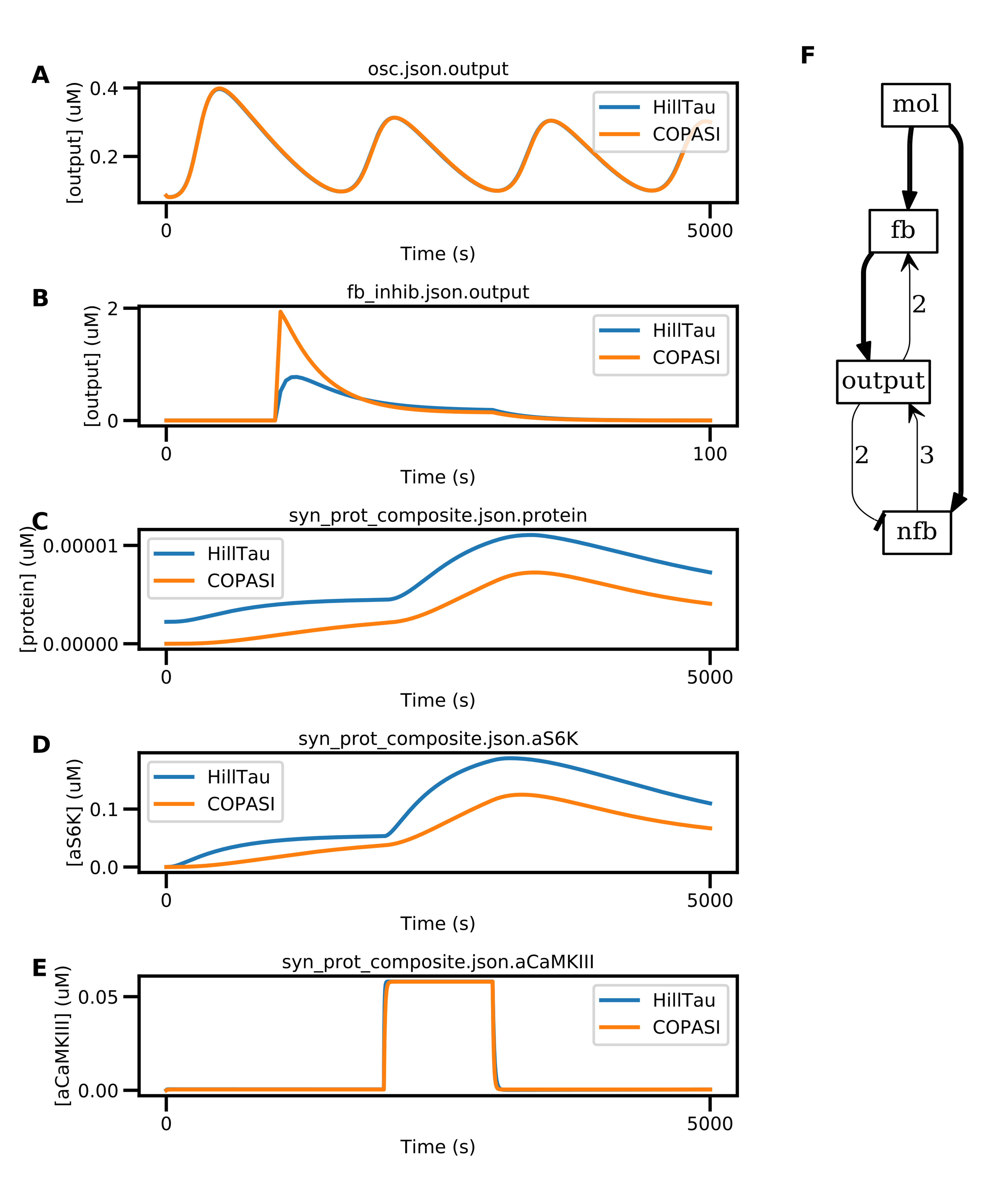

Supplement: S5 Fig — A: Oscillator model. This uses only ‘tau’ in its formulation, and fits to within 1%. B. Feedback inhibition model from Fig 2. A 1 uM stimulus is delivered at t = 20, and it lasts till t = 60. This has a mediocre fit of 29%. C-E: Protein synthesis model. C. Comparing protein synthesis response to a BDNF stimulus of 5 nM from t = 2000s to t = 3000s. Fit = 30% is mediocre. D. S6K activation in response to a BDNF stimulus of 5 nM from t = 2000s to t = 3000s. Fit = 26% is mediocre. E. CaMKIII activation in response to a calcium stimulus of 5 uM from t = 2000 to t = 3000s. This fits well, 1.7%. F. HillTau reaction scheme for oscillator model. (TIF) [file pcbi.1009621.s005.tif]

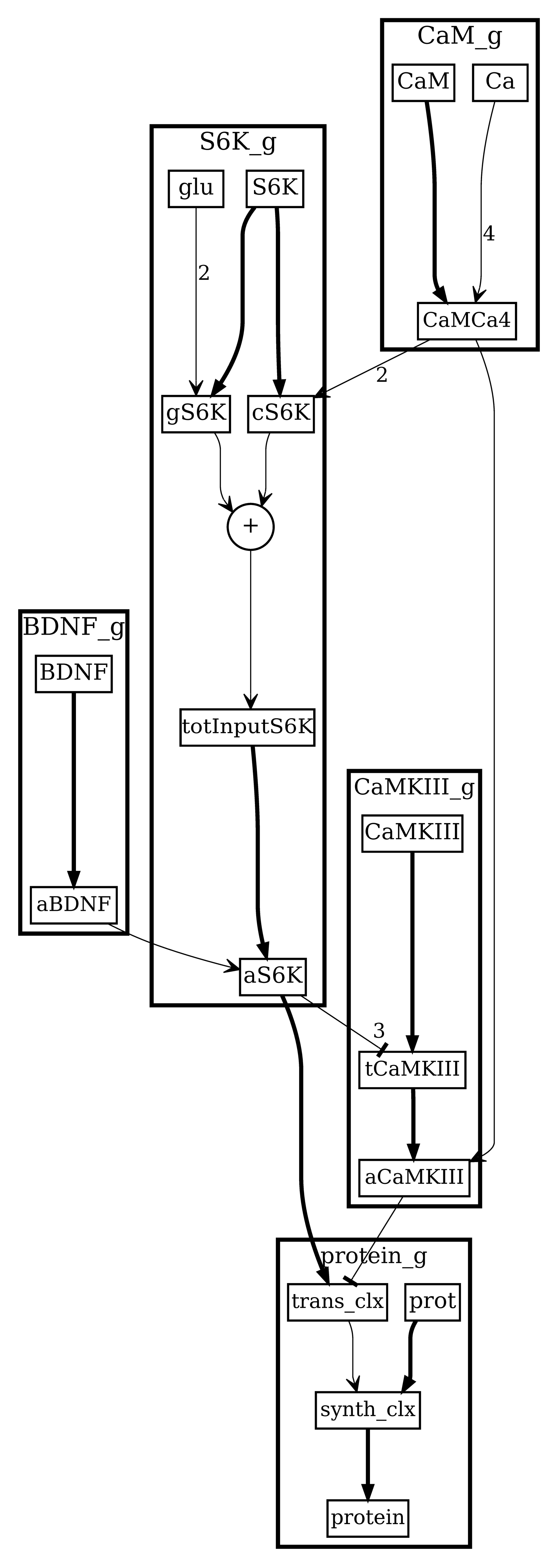

Supplement: S6 Fig — (TIF) [file pcbi.1009621.s006.tif]

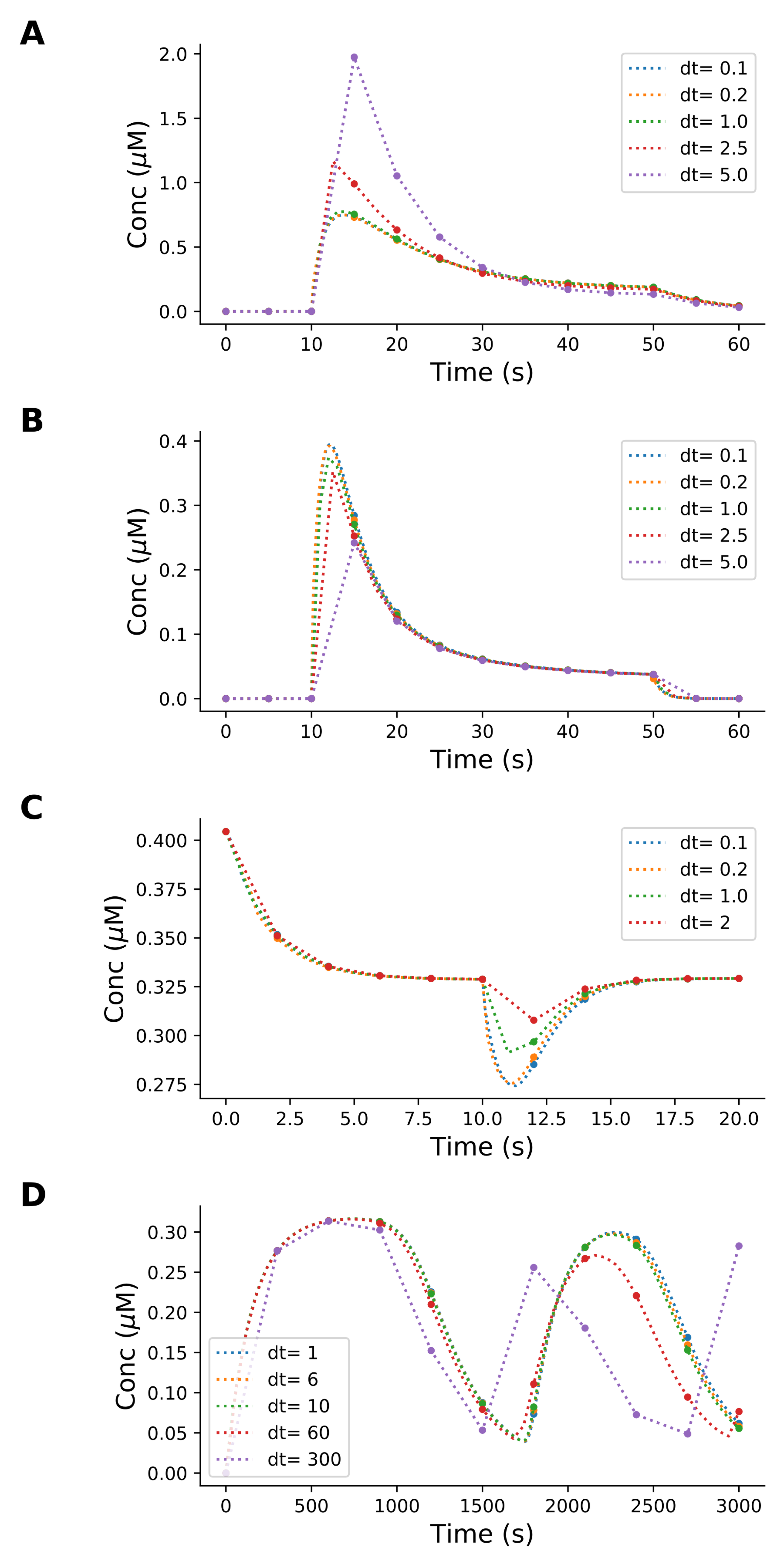

Supplement: S7 Fig — In all panels the dashed lines represent the time-series, and the dots represent the sample points for estimating error using the smallest timestep as reference. Accuracy is reported as normalized root-mean square difference from smallest timestep. A: Feedback inhibition. Step stimulus of 1 uM is given at t = 10s, which lasts till t = 50s. 1% accuracy is achieved for dt = 1s. B: feedforward inhibition. Stimulus same as A. 1.5% accuracy at dt = 1s. C: BCM curve. Stimulus of 1 uM is given at t = 10s and stays till the end of the simulation. 1% accuracy at dt = 1s. D: Kholodenko oscillator. Here the system is free-running. 1.2% accuracy at dt = 6s. (TIF) [file pcbi.1009621.s007.tif]
