# Supplementary material for: HillTau: A fast, compact abstraction for model reduction in biochemical signaling networks
Source: PLoS Comput Biol. 2021 Nov 29;17(11):e1009621. doi: 10.1371/journal.pcbi.1009621 (PMC8659295; doi:10.1371/journal.pcbi.1009621)
Supplement: Attachment — Submitted filename: ResponseToReviewers.pdf [file pcbi.1009621.s009.pdf]

I thank the editor and reviewer for these helpful comments. I have addressed all the points, as detailed below. My responses are in green and excerpts from the MS in red.

Reviewer #1: Report on the paper "HillTau: A fast, compact abstraction for model reduction in biochemical signaling"

This paper introduces a new methodology and tool for simulating signaling networks. I find the approach an interesting alternative to mechanistic ODE models. As the author clearly points out, HillTau has less parameters than mechanistic models and the execution time is several orders of magnitude faster. These crucial advantages are appropriately exploited by the machine learning features of the present tool.
Thank you

However, I find the presentation of the tool and methodology rather expeditious. The mathematical foundations are far from clear and may lead to confusion.
The revision addresses these points. The first section of Results now includes a much expanded introduction to the methodology, including equations to introduce the mathematical basis. The details on the calculations are provided in the methods section.

The relation with other abstraction methods is also not clearly presented.
This has been considerably expanded in the introduction (around line 74-76, and also lines 82-90) and also in the discussion.

Given the added value of the tool and methodology I recommend publication, provided that the following points are clarified:

1) Introduction.
a) The most current approach in machine learning of dynamical signaling networks is based on neural network type models employing sigmoidal functions (see for instance Nyman et al Plos Comp Biol 2020, PMID: 32667922). A parallel/comparison to these approaches should be drawn.
This is a useful reference, and I have added a paragraph around this, and also included earlier references.
One frequently used form specifies rate of change of concentration of each molecule as a weighted sum of input molecule concentrations, which may be passed through a sigmoid to achieve saturation (Savageau 2001; Nyman et al. 2020; Bray 1995). This form is quite similar to neural network models. Thus it lends itself to machine learning approaches to obtain parameters from systematic experimental time-series measurements (Nyman et al. 2020). The authors obtained relatively sparse interaction weight matrices, thus keeping down the number of parameters. While this formulation is effective at modeling dynamics of molecules in reaction networks, the resultant interaction matrices do not map directly to reaction pathways.

b) The fast reaction detection invoqued at line 72 is a particular case of more general quasi-equilibrium and quasi-steady state based model reduction methods reviewed in Radulescu et al Frontiers in Genetics 2012, PMID: 22833754. These reduction methods use graph rewriting and retain indeed the chemical reaction network formalism as

mentioned at line 76.
Thank you for this useful reference, the citation and text have been updated to refer to it.
These are a subset of general approaches to model reduction using quasi-equilibrium and quasi-steady state methods (reviewed in (O. Radulescu et al. 2012))

2) Hilltau schemes.
The figures and examples are useful for understanding what a Hilltau model is, but are not sufficient. I was not able to find in the paper an algorithmic description about how to generate a Hilltau scheme given a full mechanistic model. Are these abstractions obtained manually?
The approach is outlined in Figure 4 and is now stated in an substantially expanded section under “HillTau models can be optimized to fit biochemical measurements.” After detailing the steps for generating a HillTau model, we state:
Note that in principle many of these steps can be automated. For example, one can generate a family of models algorithmically (e.g., Ramakrishna and Bhalla 2008) and optimize over topology as well as parameters, but this is out of the scope of the current study. Other algorithmic approaches for model reduction are discussed in (O. Radulescu et al. 2012). In the current paper we have used the above heuristics to generate HillTau schemes (model topology) by hand.
Further, we illustrate the model development process in an extended example on synaptic activity driven protein synthesis, around Figure 4 and Figure 5.
In addition, we include a paragraph describing how the MAPK oscillator model was constructed and parameterized, around line 200.

It would be also interesting to discuss the generation of Hilltau models starting with protein-protein interaction networks that are available in larger quantity and scale than the mechanistic models.
This is an interesting suggestion and I have added a section in the discussion to consider how to utilize such interaction networks. This is something I would like to follow up in future work.

3) Testing accuracy.
The author has presented the results of extensive benchmarking of his tool. However, I would have liked to see extensive data not only on the efficiency (Figure 6) but also on the accuracy of HillTau.
This is a useful suggestion. I now include a discussion on accuracy in the text, and I have added a new supplementary figure (reproduced below) to look at how accuracy scales with timestep. In brief, for the time-series calculations we used a timestep of 1 second, which usually gave accurate results to within 1%. We further implemented an automated timestep assignment algorithm in the HillTau code, and will continue to refine this.
...on each timestep the updates are carried out in an order which ensures that inputs ripple in order through the cascade of reactions. This may lead to inaccurate estimation of transient responses if the updates are carried out at greater intervals (timestep Δt) than the time-course (τ) of the fastest reaction in the model (Supplementary Figure S7). To address this, HillTau assigns an internal timestep Δt which is smaller than the smallest τ in the model.

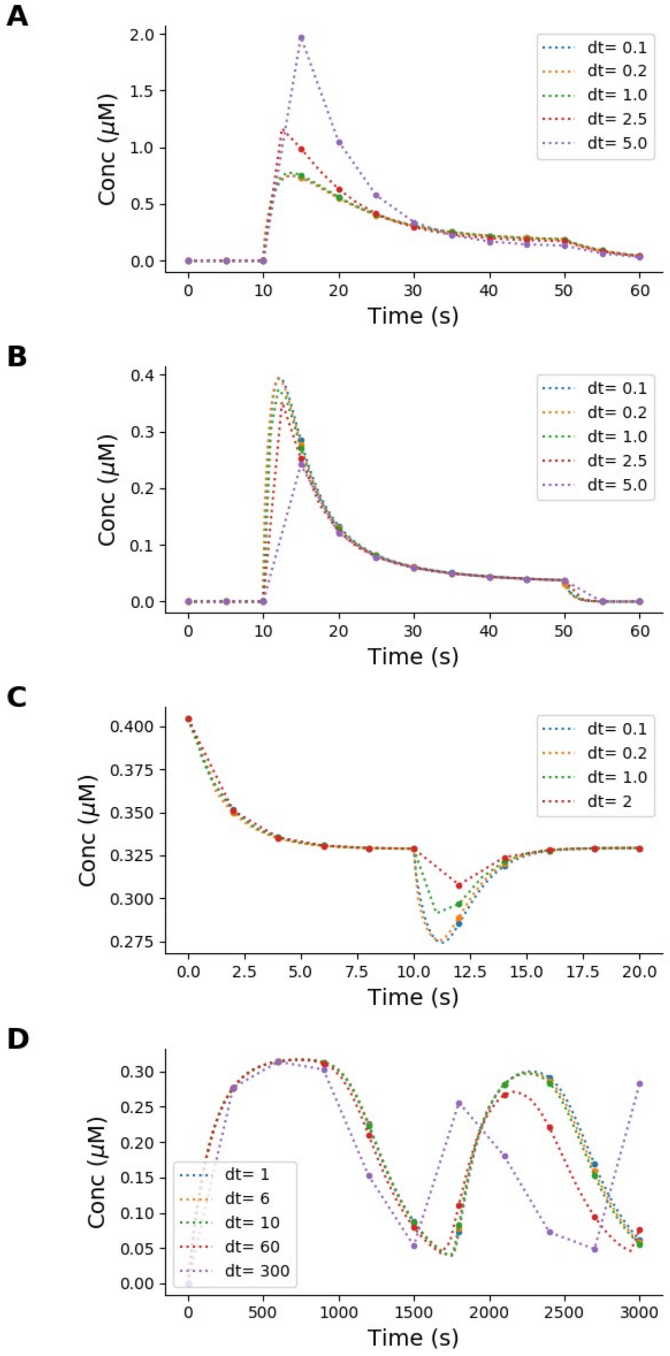

Supplementary Figure S7. Dependence of HillTau simulation output on timestep. In all panels the dashed lines represent the time-series, and the dots represent the sample points for estimating error using the smallest timestep as reference. Accuracy is reported as normalized root-mean square difference from smallest timestep. A: Feedback inhibition. Step stimulus of 1 uM is given at t = 10s, which lasts till t = 50s. 1% accuracy is achieved for dt=1s. B: feedforward inhibition. Stimulus same as A. 1.5% accuracy at dt=1s. C: BCM curve. Stimulus of 1 uM is given at t = 10s and stays till the end of the simulation. 1% accuracy at dt=1s. D: Kholodenko oscillator. Here the system is free-running. 1.2% accuracy at dt=6s.

4) Domain of validity.
Several properties of mechanistic models are necessarily lost by this approach. For instance, even if multistationarity with
feedback can be reproduced, multistationarity without feedback (resulting from sequestration effects, see for instance Markevich et al J Cell Biol 2004
PMID: 14744999) is lost. There is a bit of confusion here concerning conservation of the steady states. The method conserves steady states
to some extent, but not stoechiometry classes; this subtle difference leads to the lost of multistationarity without feed-back.
It would be useful that the author warns about other properties that may be lost by using HillTau abstraction.
It is correct, as the reviewer points out, that some properties of mechanistic models are lost because the HillTau approximation is entirely feed-forward. There is a section in the discussion which indicates the kinds of cases where HillTau approximations must be made with care for these reasons.
HillTau supports explicit cross-talk interactions, but does not introduce implicit interactions. In this regard it differs from mass-action reaction systems, in which mechanisms such as back-reactions or enzyme sequestration may introduce subtle effects, such as implicit feedback. For example, one can achieve bistability through multistage phosphorylation/dephosphorylation cascades (Markevich, Hoek, and Kholodenko 2004). To represent such effects in HillTau one would have to introduce explicit feedback steps between reactions, such as in the bistability example in Figure 2. Similarly, interesting behavior emerging from chemical saturation, such as zero-order ultrasensitivity (Goldbeter and Koshland 1981) would require the use of the explicit math expressions supported by HillTau.

5) Methods.
a) definitions should be provided for different concepts, for instance input concentration to a signaling step, level of a signaling step, ligand, modifier, etc.
The first section in Results is now expanded with equations and new explanations of these concepts.
b) It is not clear why Eq 1.7 does not admit modifiers (line 599)
The statement was incorrect. I have fixed it:
This form is completely general and any combination of inputs, interaction functions and modifiers can be implemented through suitable expressions.
c) rate interpretation of HillTau (lines 637 - 670). I could understand this heuristics, but here one should notice that the ligands and modifiers are considered
constant in the rate interpretation. This assumption may work for small time steps, but not for large time steps. The author seem to care about the size
of the time step only in the presence
of feedback, whereas large time steps may lead to inacurrate dynamics also without feedback. The main issue here is that the dynamics of one variable in a
network is not mono-exponential, but multiexponential. Only when the timescales of the variables are well separated, single variables have mono-exponential
dynamics. This remark can be related to the previous point 4).
This point is well taken and has been addressed above following point 3, testing accuracy.

I have now incorporated an automatic timestep assignment routine. It finds the smallest time-course tau in the system, and assigns a timestep 5 times smaller. This is over-cautious and will be optimized later. I have also updated the benchmarks, which turn out to be sensitive to the time-step in some cases.

d) motivation for the HillTau formalism (lines 688-747). This part of the methods should be entirely revisited as it is neither clear nor rigorous.
The methods section has been updated to explain this more carefully.
The initial motivation for the HillTau form was the observation that many stimulus-response curves in signaling have a saturating, Hill-like concentration dependence on input strength even if there are multiple intermediate steps (U S Bhalla and Iyengar 1999; Jain and Bhalla 2009). Further, many stimulus-response time-courses are visually similar to exponential time-courses. This suggested that a combination of these two properties might be a good approximation even to multi-step signaling cascades.
In order to mathematically support this idea, we considered two of the common motifs in signaling: enzyme-activation of molecules such as phospho-proteins, with a balancing deactivation reaction; and binding of activators to a reagent. Below, we show that the HillTau form achieves a reasonable approximation both to the amplitude and time-course of the response.
Following this section I provide a mathematical examination of enzyme cascades and binding reactions to show that both fit in the HillTau formulation. I have added explanatory text to clarify the application of the equations.

Reviewer #2: The author proposes a new coarse grained modeling approach using equations the author calls HillTau which incorporates Hill like equations coupled to an exponential term to model time delays. There could merit in this approach as there is a significant problem in being able coarse grain signaling models and a solution to this problem is needed.
Thank you, this is indeed one of the goals of this project.

I would like to see one thing added to the paper near the beginning which is a step-by-step example of how someone could take, say the MAPK pathway with feedback and convert it into a HillTau model.
I have now added a paragraph which outlines the process by which the MAPK HillTau model was generated. In addition, I have already provided a detailed stepwise account of the construction of the model of synaptic protein synthesis which forms the basis for Figure 4 and 5.

I note that the author has conveniently provided two python utilities to convert to and from HillTau models and SBML. I wonder if the simulation tooling the author provides is even needed because once in SBML it can be modeling by many other applications, but that is more of an observation than anything else.
The HillTau form is much faster than ODE solvers, and for this reason this simulation tooling is already in production use in our lab.

Finally, the authors states:
“It is also possible to use linear algebraic root-finding to find the steady-state value in one step, but this is prone to numerical challenges even in modern simulators like COPASI”
I don’t believe this. The author cites Clarke 1981 to support this claim but since that publication a lot of work has been done on the problem and so long as the initial starting point is not too far away from the solution, using newton solvers to find the steady state

invariably works. NLEQ and Kinsolv are two very robust newton solvers. I agree that problems will arise if there are conserved moieties in the network which are common in signaling pathways. However, this is easily taken care of by doing a row reduction of the stoichiometry matrix first. One can even do a short presimulation (which many of the mainstream simulators do) to give the solvers a good starting point. I am happy to be corrected if the author can find more recent publications that suggest there are problems.
This is a valid point and I too have looked for more recent publications on this approach. I was reporting the situation based on my experiences with implementing root finding in MOOSE, and on the COPASI simulator. While I am sure that there are other implementations which may address these concerns, I encountered a couple of kinds of problems. First, when the model system includes mathematical expressions (for example for fast equilibria or summing molecule concentrations) this seems to pose numerical difficulties. Second, the default initial control parameters for root finding did not converge in a test case, and indeed other users had also reported similar challenges, which the COPASI team very helpfully provided suggestions to work around. In this revision I have rephrased the sentence and added a more recent citation to nonlinear root-finders by Hindmarsh et al.
It is also possible to use linear algebraic root-finding to find the steady-state value in one step, possibly following a short presimulation to bring the system closer to the steady-state (Clarke, Bruce L. 1981; Hindmarsh et al. 2005).

Reviewer #3: This manuscript describes a framework, dubbed HillTau for representing reaction-diffusion systems that is at once both familiar and novel. The framework is familiar in that it is based on Hill equations for steady states and biologically meaningful time constants. The novelty lies in the event-based simulation this approach enables, as the time of the next transition can be calculated using simple arithmetic expressions. This approach vastly reduces the number of parameters relative to mass-action kinetics by avoiding the need to simulate every interaction and instead focus on the relationships between key concentrations. The potential benefit from reducing the number of parameters and avoiding the need for integration through continuous time is vast as this will allow models to incorporate reaction-diffusion based kinetics at scale with minimal computational overhead. The discussion highlights four potential areas of application: model reduction, system abstraction, scaffolds for model fitting, and efficient approximations to complex signaling.

I think this is mostly a very promising paper with an associated well-documented GitHub repository, however I do have two larger concerns: (1) I'm not sure how we're supposed to interpret quality of fit, (2) the order of the paper was confusing with the HillTau model not being explained or motivated until the methods section.

With respect to quality of fit, the main metric used here is RMS error, which gives promising values, but visually the orange (HillTau) and blue (original) look fairly different to me in Figures 3C, 3D, 5D-G, and 5J. In 5G, there is a systematic bias, with the HillTau solution always below the mass-action version. Curiously, there is even visible difference in 2C, which has fairly smooth kinetics. To the extent that the output of these kinetics may feed into some non-linear system, these variations could be significant. The fits may very well be good enough for modeling, but this is not currently self-evident.
The reviewer's point is well taken. HillTau trades accuracy for compactness, and this leads to these differences.
The reviewer has correctly noted that there seems to be a systematic bias in Figure 5G, and also a similar one in F. These emerge from the lower steady-state response of the HillTau model at low BDNF values (e.g, Figure 5I). Additional parameters or reactions

could address this, and it too comes under the overall tradeoffs. I have discussed these differences explicitly in the text, explaining the tradeoffs.
At this stage one could choose to perform further optimization, in a couple of ways. We could have obtained closer fits had we optimized to the same stimuli as in the figure panels. Instead we used more general input time-series and dose-response stimuli to the MASH optimizer to see how well the model would generalize. This gives a HillTau model that behaves well across a wider range of conditions than the experiments in figure 5. Second, there is a small systematic difference in baseline in panels 5F and 5G arising from a difference in output at resting BDNF, seen in panel 5I. The introduction of additional intermediate reactions in the model as per Figure 4 could further improve the fit. Such tradeoffs between generality, accuracy, and model complexity are common, and given that HillTau is meant for building compact models we considered this fit sufficiently good for illustration.

As far as the order goes, the results section opens with a brief description of HillTau, but until getting to the methods, there was no concrete example or equation, making it hard to understand what exactly the approach is that's being described and how it allows event-based simulation.
The first section of results has now been expanded and introduces the equations right away.

The multiple time constants mentioned in line 326 had not been previously mentioned, leading to additional confusion about the nature of the method.
This is also now introduced in the first section of results.

All-in-all, the paper does a good job describing the method, but it could benefit from some of that description and motivation happening earlier. Perhaps the HillTau formalization could be thought of as a result, with the mehtods being mostly e.g. how to fit?
Done, as mentioned above.

Minor:

It is not clear what the numbers in e.g. Figure 2F and 2G (and similarly elsewhere) mean.
These represent the order 'n' of the activation/inhibition. It is similar to the form used in SBGN notation for stoichiometry but should have been explicitly stated. This has now been clarified in the updated initial section explaining HillTau methods.

Given that a key strength is that the time constants are supposed to be biologically meaningful, it's too bad that the models are being fit using scipy optimization. Is there a principled way to derive the time constants from the mass-action rates?
This is a helpful suggestion. One can get quite close to target time-constants from first principles, and now this is described in the much expanded section on "HillTau models can be optimized to fit biochemical measurements":

There are also simple steps to obtain initial parameter estimates for each HillTau 'reaction':
1. KA from the activator concentration at half-maximum of the experimental activation curve, or directly from mass-action model rates.
2. Time-course τ from the experimental time-course of the reaction, or from the slowest intermediate step of a mass-action model. If there is a distinct time-course when the reaction turns off, use this as τ2.

3. When the modulator is present, assign Kmod and Amod from the half-maximum and the steepness of modulation curve.

In at least some of the examples e.g. Examples/PaperFigures/bench_native.py, elapsed time is measured using deltas of time.time(), which gives timings to the nearest 1/64 sec. time.perf_counter() gives higher resolution times that are not susceptible to e.g. changes in system clock. See https://www.webucator.com/article/python-clocks-explained/ for a discussion.
Thank you for this pointer. The information provided at this link does not seem to apply to my Ubuntu 18.04 system. I wrote a script which queries the time module. This reports a 1 nanosecond resolution for all the clocks.

time namespace(adjustable=True, implementation='clock_gettime(CLOCK_REALTIME)', monotonic=False, resolution=1e-09)
monotonic namespace(adjustable=False, implementation= 'clock_gettime(CLOCK_MONOTONIC)', monotonic=True, resolution=1e-09)
perf_counter namespace(adjustable=False, implementation= 'clock_gettime(CLOCK_MONOTONIC)', monotonic=True, resolution=1e-09)
process_time namespace(adjustable=False, implementation= 'clock_gettime(CLOCK_PROCESS_CPUTIME_ID)', monotonic=True, resolution=1e-09)

I also plotted a histogram of timings for a small test script. It has some odd periodicities that crop up for perf_counter() as well as time.time(), but I don't see that there is anything to choose between them.

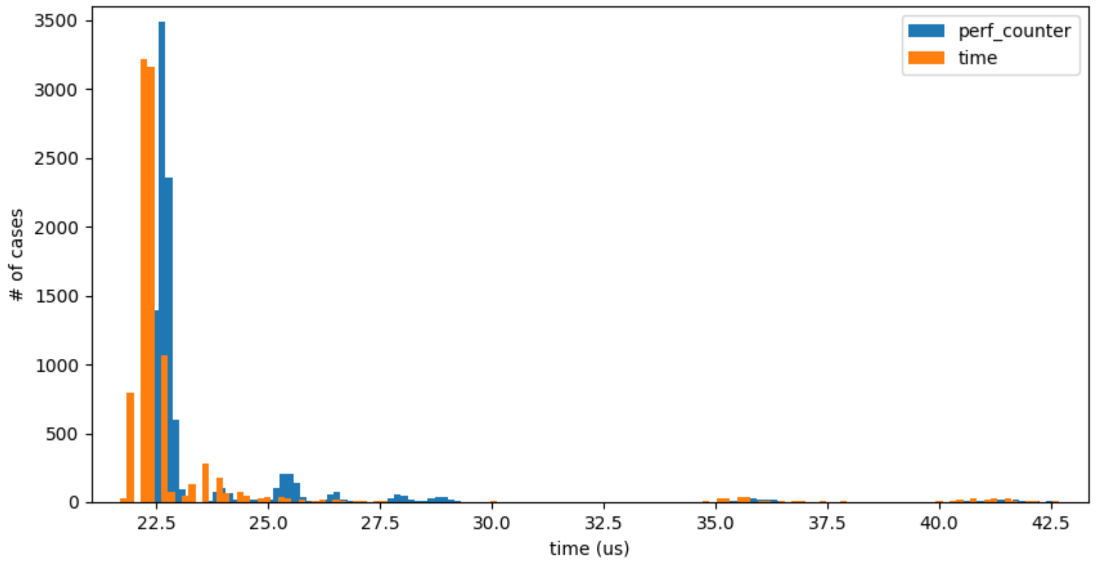

Line 345: "setused" -> "set used" (missing space)
Thank you, corrected.
