# Supplementary material for: HillTau: A fast, compact abstraction for model reduction in biochemical signaling networks
Source: PLoS Comput Biol. 2021 Nov 29;17(11):e1009621. doi: 10.1371/journal.pcbi.1009621 (PMC8659295; doi:10.1371/journal.pcbi.1009621)
Supplement: Attachment — Submitted filename: Response_v3.pdf [file pcbi.1009621.s010.pdf]

I thank the Editors and referees for the careful examination of the MS. I have addressed the remaining comments of the third reviewers.

Yours sincerely,
Upinder S. Bhalla.

Response to Referee comments.

Reviewer #1: The revised version of the manuscript addresses all my recommendations and can be accepted.

Thank you

Reviewer #2: No further comments on the paper.

Thank you

Reviewer #3: I thank the author for his responses, which largely address my concerns. This manuscript has been strengthened by his edits, especially the addition of a summary of the nature of the HillTau model at the beginning of the results section. In brief, HillTau can be viewed as a phenomenological model that lumps multiple reactions together. Key features are its event-driven nature which allows simulations to make relatively large advances as compared to differential-equation based models, and asymmetric exponential change. Feedback cycles are not directly compatible with the model, but can be approximated by breaking the cycle and using small advances.

Thank you

Although, honestly, I'm still a little confused because the approach uses an internal time-step so it can't skip from event to event.

This is a good point, I have clarified in the text around line 178.

For typical use-cases, such as synaptic plasticity models, the event interval is shorter than the time-courses in the model (typically ~1 sec) and hence only a single step is taken. In cases where HillTau inserts additional time-steps for accuracy, it is done behind the scenes of the same event-driven programming interface.

The existing implementation on GitHub is pure Python, but the readme promises a faster C++ version that preserves the existing Python API. This will be invaluable.

A mature C++ version has been on GitHub since July, but it has taken some while to implement a pip-install capability for a range of operating systems. Now this works for Linux platforms and others are in the works.

I still wonder how I would decide if a simplified HillTau model was good enough for my

purposes, but the authors points in lines 397-406 (all line numbers here and below are in the version with edits visible) do a good job of addressing the tradeoffs inherent in building "compact models."

Thank you.

Minor:

lines 183-184: A factor of 10 usually gives good convergence. <-- would benefit from knowing what evidence this was based on

I have inserted a line to explain this, with reference to supplementary figure S7 where I explore fits for a number of models including two with feedback (the feedback inhibition model and the Kholodenko oscillator model.)

A factor of 10 usually gives good convergence (e.g., Supplementary Figure S7 panels A and D).

MASH is introduced in lines 195-197 , used in 209-210, then seemingly reintroduced in 358-361... would be better if this was consolidated.

Thank you for pointing this out. I have updated the text to consolidate. Line 196 now leads into the following text, and the duplicate text below has been removed.

MASH runs the reference model through a range of stimuli designed to explore its input-output properties, and then uses numerical optimization methods from scipy.optimize to tune parameters so that the HillTau model produces a good fit to the original.

line 357: readers may not be familiar with FindSim (Viswan et al. 2018); the paper would benefit from a brief description... it's not clear why that approach is best here vs other approaches.

I agree. I have inserted the following text to explain.

In brief, FindSim provides a Python-based framework for matching models to experiments. It codifies the experiment design (e.g., time-series, dose-response, bar-chart) and experimental results into a single machine-readable file. FindSim runs the experiment on the model and returns a numerical score for goodness of fit. The model may be defined in SBML (run using MOOSE) or using HillTau. Thus FindSim can be used as the scoring function for optimizing model fit to experiments using a variety of optimization methods available in scipy.optimize.

lines 457-458: not 100% clear what you're considering state variables here; usually these would be things that have associated rates of change, and then you very much would want to know the initial values. I'm thinking of things like gating variables in Hodgkin-Huxley. There's no initial concentration but there's definitely an initial value that matters.

This is a good point. I now address it in the text:

Here we consider state variables to be those which are computed, as opposed to defined using initial conditions. In ODE models we estimate this by counting the number of rate

terms plus the number of molecular species with a non-zero initial value. In HillTau models we count the rate terms and the species which are not reaction outputs. This yields the approximate scaling terms below.

Line 469: mysterious space in the middle of C++.

Fixed.

Line 914: I think "HillTaul" is a typo, otherwise I missed something major.

Fixed

In addition to the above changes, I have updated the benchmark plots in Figure 6 to reflect further improvements to the automated timestep algorithm in HillTau. The regression fit scores in the figure 6 legend and text have been updated to match.
